# Topical Rosiglitazone Is an Effective Anti-Scarring Agent in the Cornea

**DOI:** 10.1371/journal.pone.0070785

**Published:** 2013-08-05

**Authors:** Krystel R. Huxlin, Holly B. Hindman, Kye-Im Jeon, Jens Bühren, Scott MacRae, Margaret DeMagistris, David Ciufo, Patricia J. Sime, Richard P. Phipps

**Affiliations:** 1 Flaum Eye Institute, University of Rochester, Rochester, New York, United States of America; 2 Center for Visual Science, University of Rochester, Rochester, New York, United States of America; 3 Department of Ophthalmology, Goethe University, Frankfurt am Main, Germany; 4 Department for Environmental Medicine, University of Rochester, Rochester, New York, United States of America; 5 Department of Medicine, University of Rochester, Rochester, New York, United States of America; Cedars-Sinai Medical Center, United States of America

## Abstract

Corneal scarring remains a major cause of blindness world-wide, with limited treatment options, all of which have side-effects. Here, we tested the hypothesis that topical application of Rosiglitazone, a Thiazolidinedione and ligand of peroxisome proliferator activated receptor gamma (PPARγ), can effectively block scar formation in a cat model of corneal damage. Adult cats underwent bilateral epithelial debridement followed by excimer laser ablation of the central corneal stroma to a depth of ∼160 µm as a means of experimentally inducing a reproducible wound. Eyes were then left untreated, or received 50 µl of either 10 µM Rosiglitazone in DMSO/Celluvisc, DMSO/Celluvisc vehicle or Celluvisc vehicle twice daily for 2 weeks. Cellular aspects of corneal wound healing were evaluated with *in vivo* confocal imaging and post-mortem immunohistochemistry for alpha smooth muscle actin (αSMA). Impacts of the wound and treatments on optical quality were assessed using wavefront sensing and optical coherence tomography at 2, 4, 8 and 12 weeks post-operatively. In parallel, cat corneal fibroblasts were cultured to assess the effects of Rosiglitazone on TGFβ-induced αSMA expression. Topical application of Rosiglitazone to cat eyes after injury decreased αSMA expression and haze, as well as the induction of lower-order and residual, higher-order wavefront aberrations compared to vehicle-treated eyes. Rosiglitazone also inhibited TGFβ-induced αSMA expression in cultured corneal fibroblasts. In conclusion, Rosiglitazone effectively controlled corneal fibrosis *in vivo* and *in vitro*, while restoring corneal thickness and optics. Its topical application may represent an effective, new avenue for the prevention of corneal scarring with distinct advantages for pathologically thin corneas.

## Introduction

Damage to the mammalian cornea causes a rapid cellular reaction, which attempts to heal the wound. Disruption of the epithelial layer, basement membrane and injury to stromal keratocytes release a range of cytokines, including transforming growth factor β (TGFβ) - probably the strongest pro-fibrotic agent [1]. These cytokines stimulate epithelial regeneration, keratocyte proliferation [2], [3], migration [4], differentiation into fibroblasts and myofibroblasts [3], the deposition of abnormal extracellular matrix (ECM) proteins and the recruitment of macrophages and other immune cells into the cornea – reviewed in [5], [6]. When stromal keratocytes are stimulated by TGFβ to differentiate into myofibroblasts [6], they assemble f-actin into stress fibers and express α-smooth muscle actin (αSMA) [7], [8], which endow them with contractile properties [3], [9]. Though undoubtedly useful for the goal of shrinking the injury site, wound contraction in the cornea disturbs the shape and curvature of this critical optic and its ability to precisely focus light onto the retina. As a result, corneal wounds increase the optical aberrations of the eye, decreasing visual quality in a manner that is separate from (but adds to) that caused by loss of transparency.

Loss of transparency, largely in the form of haze during corneal wound healing results from several factors: 1) infiltration of the cornea by inflammatory cells, 2) αSMA positive myofibroblasts, which are less transparent than quiescent keratocytes, likely due to their decreased crystallin synthesis [10], [11], and 3) myofibroblasts laying down ECM that is both differently organized and composed of molecules (including collagen III, hyaluronan and other proteoglycans low in keratan sulfate components) which are neither part of the normal stromal ECM, nor conducive to good corneal transparency [6], [12], [13], [14].

Clinically, attempts to control corneal scarring have mostly involved the use of steroids or Mitomycin C (MMC) [15], [16]. While effective at decreasing myofibroblast differentiation and haze [15], [17], [18], [19], [20], [21], [22], [23], [24], these two compounds exhibit significant side-effects and in the case of MMC, toxicity and DNA damage to stromal keratocytes and endothelial cells, that could bear long-term negative consequences for ocular health [25]. Experimentally, ocular application of antibodies against TGFβ after excimer laser ablation of the corneal surface reduced myofibroblast differentiation, migration and corneal reflectivity (haze) in both rabbits [26] and cats [27]. However, epithelial healing was slowed and with application for longer than 3 days, stromal regeneration was also blocked [27]. Here, we asked whether small molecule pharmacologics, capable of manipulating intracellular signaling downstream of TGFβ receptor activation might represent a better alternative to steroids, MMC and topical application of anti-TGFβ antibodies to the eye. In particular, it would be advantageous to find a means of preventing haze and myofibroblast differentiation, while stimulating regeneration of the corneal stroma and epithelium, and preserving or restoring normal ocular optics. The cat was chosen as the *in vivo* (and *in vitro*) model in the present study because it offers several advantages over rabbits and mice. First, the cat cornea bears strong similarities to the human cornea: it has similar structural organization, similar stromal and epithelial thicknesses [28], and similar corneal wound healing responses [29], [30]. Second, cats can be behaviorally trained, allowing us to reliably measure optical correlates of corneal wound healing repeatedly, over time, using the same instrument (wavefront sensor), in the same state (awake-fixating) and with the same degree of precision attained in humans [27], [31], [32].

With respect to understanding intracellular signaling downstream of TGFβ activation, this is usually studied *in vitro* using “wounding“ models in which cultured stromal fibroblasts are stimulated to differentiate into myofibroblasts with application of TGFβ to the culture medium [7], [33], [34], [35]. Here, we showed that feline corneal fibroblasts exhibit similar responses in culture as fibroblasts from other species [7], [33], [34], [35], [36]. This is a critical point since it is evidence from *in vitro* models, as well as from a few clinical and *in vivo* studies in a range of body tissues [37], [38], [39], [40], [41], [42], [43], [44], that first revealed strong anti-fibrotic properties for an interesting class of molecules, known a peroxisome proliferator activated receptor gamma (PPARγ) ligands. PPARs are nuclear receptors that function as transcription factors [37], [45] and are best known for their important roles in lipid metabolism [46], [47]. Tissue distribution of PPARγ− a subtype of PPAR - varies, with highest concentrations in adipose tissue [48], and lower, but identifiable levels in most bodily organs and cell types [44], [49], [50]. Of relevance to the present work, Saika and colleagues showed that viral transfection and over-expression of PPARγ in the living mouse cornea reduced myofibroblast differentiation, up-regulation of several cytokines and matrix metalloproteases, and macrophage/monocyte invasion following alkali burn injuries [51]. In cultured fibroblasts stimulated with TGFγ, the same authors showed that PPARγ over-expression blocked nuclear translocation of phosphorylated SMAD2, confirming that PPARγ exerted its potent anti-fibrotic effects in wounded corneas at least in part, by blocking signaling downstream of TGFβ receptor binding.

Since viral transfection is not yet widely practiced clinically (although see [52]), we asked whether similarly potent anti-fibrotic effects might be attained in wounded corneas with topical administration of PPARγ ligands/agonists – in our case, Rosiglitazone (Avandia®, GlaxoSmith-Kline). To date, with respect to corneal applications, PPARγ ligands have only been used *in vitro*, where Pioglitazone [53], 15d-PGJ2 [54] and CDDO-Me [54] appeared able to block TGFβ-induced differentiation of corneal fibroblasts into myofibroblasts. The present study is the first to administer a PPARγ ligand topically to the living eye following laser ablation of the stroma. Our goal was to assess the impact of prolonged administration of this drug on corneal scarring, tissue regeneration and optical quality *in vivo*. Excimer laser ablation was used here because its consequences for the cornea have been well described in the clinical and experimental literature [27], [55], [56], [57], [58]. For experimentalists, excimer laser ablations represent an excellent means of reproducibly inducing a corneal wound with a defined shape, size and depth, and with predictable biological and optical consequences – reviewed in [59], [60], [61].

Here we report that topical Rosiglitazone reliably and effectively prevented myofibroblast differentiation, haze induction and large wavefront changes after laser ablation of the cat cornea *in vivo*. On the other hand, the epithelium regenerated fully within a normal time-frame, as did most of the stroma removed by the laser. Cell culture experiments suggested that this could be due to a previously unreported, differential effect of Rosiglitazone on αSMA and Collagen I expression following TGFβ stimulation. Three months post-operatively, Rosiglitazone-treated corneas looked morphologically and optically, almost as if they had never undergone stromal ablation.

## Materials and Methods

### Ethics Statement

All animal procedures were conducted according to the guidelines of the ARVO Statement for the Use of Animals in Ophthalmic and Vision Research, and the NIH Guide for the Care and Use of Laboratory Animals. The protocol was approved by the University of Rochester Committee on Animal Research (UCAR – Assurance number: A-3292-01).

### *In vivo* Experiments

Domestic short hair cats (*felis cattus*) underwent bilateral laser ablation followed by treatment with Rosiglitazone in dimethylsulfoxide (DMSO)/Celluvisc (RefreshCelluvisc™, Allergan; N = 8 eyes), DMSO/Celluvisc (N = 12 eyes) or Celluvisc (N = 10, from prior study from our group [27]). Details about the exact makeup of these solutions is provided below. An additional 6 eyes from 3 cats received no eye drops after bilateral laser ablation and were designated as “untreated”. One Rosiglitazone-treated eye, two Celluvisc treated eyes and two untreated eyes developed central corneal abrasions post-operatively, largely as a result of rubbing, and were excluded from all analysis. All other eyes underwent imaging at 2, 4, 8 and 12 weeks post-operatively. Sets of cats were sacrificed for histology at the 2 and 4 weeks time-points. Remaining cats were imaged at 8 and 12 weeks, then sacrificed at the 12 weeks time-point. Only a subgroup of the animals were behaviorally trained for wavefront sensing, giving us 4 eyes per treatment group in which wavefront measures were carried out at each time-point of interest. Procedures and treatments are summarized in Table 1.

**Table 1:** Number of eyes that underwent laser ablation, followed by OCT imaging, confocal imaging and/or wavefront sensing (WFS) at each post-operative time-point.

		10 µM Rosiglitazone	DMSO/Celluvisc	Celluvisc	Untreated (no WFS)
# eyes lasered		8	12	10	8
# eyes imaged - confocal, OCT (WFS)	2 weeks	7 (4)	12 (4)	8 (4)	6
	4 weeks	6 (4)	8 (4)	6 (4)	6
	8 weeks	4 (4)	4 (4)	4 (4)	4
	12 weeks	4 (4)	4 (4)	4 (4)	4

Note that the number of eyes used for wavefront analysis (in brackets, where applicable) is identical to the number of eyes imaged at the 12wk post-operative timepoint.

#### Laser ablation procedure

Excimer laser ablation in the form of photorefractive keratectomy (PRK) was performed in the center of each cornea following epithelial debridement. A conventional, myopic, -10 diopters (D) ablation was used over a 6 mm optical zone (OZ), with a central ablation depth of 168 µm (Planoscan 4.14; Bausch & Lomb Inc.) for all eyes treated with DMSO/Celluvisc, Celluvisc and all but 2 eyes treated with Rosiglitazone. More recently, upgrading of the laser system to Zyoptix (Bausch & Lomb Inc.) changed the stromal ablation depth to ∼135 µm for 2 of the Rosiglitazone-treated eyes and all of the untreated eyes. PRK was performed by one of three refractive surgeons (SM, HH, JB) under topical (Proparacaine 0.5%, Falcon) and surgical anesthesia (Ketamine, 5 mg·kg^−1^, Medetomidine Hydrochloride 0.04 mg·kg^−1^), using a Technolas 217 laser (Bausch & Lomb Inc.).

#### Pharmacological treatments

Immediately after laser ablation of the stroma, either Rosiglitazone or a vehicle solution consisting either of 10% DMSO/Celluvisc or Celluvisc was applied and held in place on the stromal bed using a saturated, sterile, gelatin sponge (Surgifoam™; Ethicon) for 2 minutes. Each eye then received a drop of antibiotic solution (Neomycin, Polymyxin B Sulphate, Gramicidin Ophthalmic Solution USP, Bausch & Lomb Inc.). For the following 2 weeks, each Rosiglitazone-treated cat eye received 1 drop (50 µl) of 10 µM Rosiglitazone (Cayman Chemical Company) diluted in vehicle solution consisting of 10% DMSO in Celluvisc twice per day and one drop of antibiotic daily. Pilot testing showed this dosage regimen to be well tolerated and to cause no overt corneal or ocular toxicity. Control eyes received identical amounts of either 10% DMSO/Celluvisc or Celluvisc vehicle solutions, followed by 1 drop of antibiotic daily. The Rosiglitazone vehicle contained 10% DMSO for two reasons: (1) Rosiglitazone is lipophilic and can be well dissolved in DMSO, and (2) DMSO is known to enhance drug penetration through membranes and tissues [62], an important factor here as administration continued for 1 week after epithelial closure. A separate group of cat eyes were treated with just Celluvisc, in order to control for the possible anti-fibrotic effects of DMSO. Data from some of these control eyes were reported in a previous publication from our group [27]. Finally, a set of eyes received no treatment after PRK other than daily administration of antibiotic drops (Neomycin, Polymyxin B Sulphate, Gramicidin Ophthalmic Solution USP, Bausch & Lomb Inc.). This latter set of eyes served as untreated controls for the possible biological and optical effects of Celluvisc in promoting corneal wound healing.

#### *In vivo* confocal imaging

Confocal imaging of the central cornea was performed in each eye before and 2, 4, 8 and 12 weeks after laser ablation to examine changes in the appearance of cells and of the extracellular matrix in the stroma, and to assess changes in endothelial cell density. Cats were anesthetized as for PRK and imaged using the Retina Tomographer with the Rostock Cornea Module (Heidelberg Engineering, Dossenheim, Germany). Lubricating gel (Genteal, Novartis) was placed on each eye and on the contact cap. Correct alignment was attained to the central cornea, focus was set to the epithelium and several automated volume scans, each 58 µm in depth, were performed until the endothelium became visible. Scans were recorded as digital video sequences and Image J (NIH) was used to count endothelial cells within the 400×400 µm field of view. Cell density was then expressed as the number of cells/mm^2^.

#### Optical Coherence Tomography (OCT)

A custom-built, anterior segment OCT was used to image feline corneas before and 2, 4, 8 and 12 weeks after laser ablation in order to measure changes in backscatter reflectivity (an index of haze) within the stroma, as well as changes in the thickness of the epithelial and stromal layers [32], [63], [64], [65], [66], [67], [68]. Cats were anesthetized as detailed above, lubricating gel (GenTeal, Novartis) was applied to the ocular surface, the head was stabilized and the OCT was centered on the pupil. Videos of the central 10 mm of each cornea were collected at 8 frames/sec. At least ten images were extracted at each time-point to carry out the following measurements:

For thickness measurements, custom software [68], [69] was used to obtain a normalized intensity profile across a rectangular area 105 µm wide, spanning the entire thickness of the cornea, 1 mm nasal (to avoid the specular reflection) to the middle of each image. The thickness of the epithelium and stroma were estimated by measuring the difference between relevant intensity peaks in each profile [68], [69].

Backscatter reflectivity was computed from 4 sampling lines in each of 10 corneal images/eye/timepoint. Of the 4 sampling lines, 2 were on each side of the central pixel of each image, starting about 1 mm from the corneal center, and with each pair of lines further separated by ∼1 mm. A pixel intensity profile from epithelium to endothelium was created for each line (Image J, NIH) before averaging across all 4 lines. To compensate for fluctuations in laser strength, this average profile was normalized to the mean pixel intensity in a background region (exterior to the cornea) in each image. The region of the curve corresponding to the stroma was then divided into thirds to compute the average, normalized pixel intensity over the anterior and posterior thirds of the cornea in each image. Normalized pixel intensity values over the anterior and posterior thirds of the cornea were then averaged across 10 images/eye/timepoint.

In all cases, measurements collected from OCT images were localized within the laser ablation zone in each eye.

#### Wavefront analysis in awake, fixating cats

Wavefront measurements were performed pre-operatively and 2, 4, 8 and 12 weeks post-PRK with a custom-built Hartmann-Shack wavefront sensor, in order to quantify changes in ocular wavefront aberrations induced by both the laser ablation and pharmacological treatments administered post-operatively. Six of the cats (12 eyes) used in this study (2 in each of the Rosiglitazone, DMSO/Celluvisc and Celluvisc treatment groups) were behaviorally trained to fixate on single spots of light presented on a computer monitor as previously described [27], [32], [70]. The sensor was aligned to the visual axis of one eye with a pupil camera, while the other eye fixated a spot of light presented on a dark computer monitor [27], [32], [70]. One hundred to two hundred video frames of each eye’s spot array patterns were collected at each time-point. Twelve patterns were analyzed per eye and time-point, and wavefront errors were calculated using a 2^nd^–10^th^ order Zernike polynomial expansion according to published standards for reporting aberration data of the eye [71]. The measurements were centered on the ablation optical zone by shifting a 6 mm centroiding area (analysis pupil) manually to find the wavefront that yielded the most negative defocus (j = 4) value, *i.e.* the maximal treatment effect [72]. For calculation of preoperative wavefront aberrations, the analysis pupil was shifted according to the mean post-operative offset relative to the pupil center. The following root mean squares (RMS) were also calculated at each time-point, ultimately being expressed in terms of magnitude change relative to pre-operative values in order to compensate for differences in baseline between animals: lower order RMS (LORMS) for j = 3 to 5; astigmatism RMS for j = 3,5, 11, 13, 23, 25, 39, 41, 59 and 61; higher-order RMS (HORMS) for j = 6 to 65; coma RMS for j = 7,8,17,18,31,32,49 and 50; spherical aberration RMS for j = 12,24, 40, 60; and residual RMS for all non-coma and non-spherical higher-order aberrations.

#### Immunohistochemistry

Following euthanasia, corneas were excised and drop-fixed in 1% paraformaldehyde/0.1 M phosphate buffered saline (PBS), pH 7.4 for 10 min. They were then transferred to 30% sucrose/0.1 M PBS, and stored at 4°C for 2 days. After embedding into blocks (Tissue Tek® O.C.T. Compound, Sakura Finetek), serial 20 µm-thick cross-sections were cut on a cryostat (2800 Frigocut E™; Leica), mounted on microscope slides and stored in a –20°C freezer until ready to stain.

Slides containing 3 corneal sections each were air dried and rinsed in 0.01 M PBS. Two sections per slide were incubated overnight at 4°C with 2 µg·mL^−1^ mouse monoclonal anti-αSMA antibody (clone 1A4, Sigma Aldrich). The third section was incubated with 0.1 M PBS as a negative control. After washing off the primary antibody with 0.01 M PBS, sections were reacted with anti-mouse IgG tagged with AlexaFluor® 488 (2 µg·mL^−1^, Molecular Probes), followed by propidium iodide (0.1 µg·mL^−1^, Invitrogen) to label cell nuclei. Double-labelled sections were imaged using an Olympus AX70 fluorescence microscope and photomicrographs were collected via a high resolution-video camera interfaced with a PC running the ImagePro software (MediaCybernetics).

### *In vitro* Experiments

#### Isolation and culture of primary feline corneal fibroblasts

Whole eyeballs were excised immediately post-mortem from young, adult domestic short-hair cats (*felis cattus*) and placed into Optisol-GS (Bausch & Lomb, Inc.). The corneal epithelium was scraped off prior to dissection of the cornea just inside (and excluding) the limbus, after which the endothelium was also removed by scraping. The isolated corneal stroma was then washed with DMEM/F12 (Cellgro™) with 1% Penicillin/Streptomycin (P/S, Gibco), rinsed with sterile balanced salt solution enriched with bicarbonate, dextrose, and glutathione (BSS plus, Alcon), and placed into 5 mg/ml Dispase II (Roche Diagnostics) in DMEM/F12 with 1%P/S overnight at 4^o^C. The next day, after mild shaking for 30 mins at 37°, the supernatant was discarded and the Dispase-treated tissue was incubated in 1 mg/ml Collagenase (*Clostridium histolyticum*, C8176, Sigma-Aldrich) with DMEM/F12 with 1%P/S at 37^o^C for 45 min. The digested stromal material was centrifuged at 700 rpm for 2 min to remove undigested tissue. The resulting supernatant, containing mostly stromal fibroblasts, was centrifuged again at 1500 rpm for 5 min and the pellet resulting from this latest round of centrifuging (presumably pure fibroblasts) was re-suspended in 2 ml of Fibroblast Growth Factor (FGF)-containing medium (C-23010, PromoCell GmbH). The number of cells obtained was counted and the cells were seeded onto culture plates (Cat.#628160, Greiner Bio-One). Two eyeballs yielded approximately 1×10^5^ primary corneal fibroblasts. The cells were grown in FGF medium, refreshed every second day. After the second passage, the medium was changed to DMEM/F12 with 5% horse serum (HS - cat.#P5552, Sigma Aldrich) and refreshed every second day until passages 6 or 7, at which point the cells were used for the experiments below, which were performed in triplicate.

#### Effect of rosiglitazone on TGFβ-induced expression of αSMA and collagen I in feline corneal fibroblasts

Passage 6–7 feline, corneal fibroblasts were seeded at a density of 2.5×10^4^–5.0×10^4^ cells/well in 6-well plates containing DMEM/F12 containing 5% HS. After attachment, which usually took 1–1.5 hrs, the medium was changed to one containing DMEM/F12 with 1% HS for 1day in order to promote cellular quiescence. Cells were then pre-treated with Rosiglitazone in 1% HS in DMEM/F12 medium for 30 min. After this time elapsed, TGFβ1 (1ng/ml – R&D Systems Inc.) was added to the medium and the cells were incubated for up to 3 days (cells were prepared for western blotting after 1, 2 or 3 days of incubation) without any medium changes. Cells were washed twice with 1x Dulbecco’s Phosphate Buffered Saline (DPBS, calcium/magnesium free, Cellgro). RIPA lysis buffer (50 mM Tris-cl pH 7.4, 150 mM NaCl, 1% NP40, 0.25% Na-deoxycholate, 1 mM sodium orthovanadate, 1 mM PMSF, 1x complete mini protease inhibitor cocktail ) was then added to the cells for 15 mins on ice, after which the lysates were collected and centrifuged for 10 mins at 12,000 RPM. The pellets were discarded and the protein concentration of residual lysates was determined using a Micro BCA kit (Bio-Rad).

In the present experiments, western blots were used to detect the expression of αSMA and Collagen I relative to the expression of tubulin. An 8% gel was run using 3–5 µg of cell lysates separated by electrophoresis, transferred to nitrocellulose membrane. Primary antibodies (mouse monoclonal anti-αSMA, 1∶10,000, Thermo Scientific; anti-tubulin, 1∶5,000, Santa Cruz; anti-Collagen I (LF-68), 1∶5,000 gifted from Dr. Larry Fisher, National Institute of Dental and Craniofacial Research, Bethesda, MD) were incubated overnight at 4°C. Secondary antibodies (anti-mouse IgG or anti-rabbit IgG-horseradish peroxidase, GE Healthcare) were then applied for 1 hr at room temperature. Bands were detected by Western Lightning ™ plus-ECL (from PerkinElmer). Finally, the membranes were scanned with a chemi-doc machine (Bio-Rad) and the resulting images were imported into Image J (NIH) for measurement of relative expression levels. For the background intensity levels, we measured two areas (one above and one below each reactive band), and averaged them together. This background level was then subtracted from the average pixel intensity over the reactive band. The band’s net intensity value was then divided by the average net intensity value of its corresponding loading control (reacted for tubulin), and the result was further normalized to that attained following stimulation with TGFβ alone.

### Statistical Analysis

When three or more intervention groups were compared, inter-group differences in reflectivity, corneal thickness, wavefront aberrations and protein expression were compared with a repeated measures ANOVA (for repeated measures over time) or a two-way ANOVA, or a one-way ANOVA for western blot analyses. When only two groups were compared, a two-sided Student’s t-test was performed. A probability of error of *P*<0.05 was considered statistically significant. All ANOVAs were performed using the SPSS 20.0.0 software package (SPSS Inc.), while t-tests were performed in Excel (Microsoft Office).

## Results

### Topical Rosiglitazone Inhibits αSMA Induction after Excimer Laser Ablation of the Cornea

**Figure 1:**
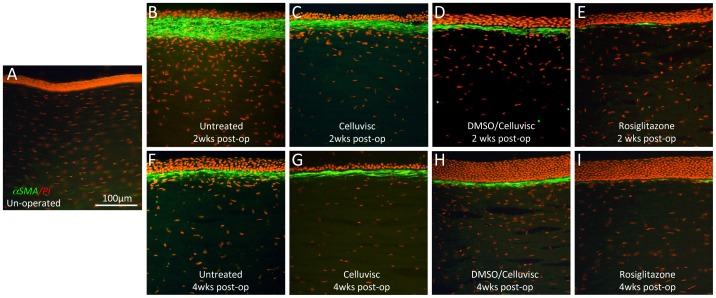
Immuno-staining for αSMA (green) in cat corneal sections counterstained with propidium iodide (PI - red). **A**. Unoperated cat cornea showing complete lack of αSMA staining. **B.** Cat cornea 2 weeks post-PRK, which received no treatment. Note the very thick band of αSMA staining just below a thin, incompletely regenerated epithelium. **C.** Cat cornea treated with Celluvisc for 2 weeks after PRK and sampled 2 weeks post-PRK. The band of αSMA staining is thinner than in B, but remains continuous and several cells thick. The epithelium is thicker than in the untreated cornea and more cell-dense. **D.** Cat cornea treated with DMSO/Celluvisc vehicle solution post-operatively, sampled at 2 weeks post-laser ablation. Note the maintained, distinct band of strong, continuous αSMA staining in the anterior stroma, just below the thick epithelium. **E.** Corresponding view of the ablation zone in a cornea treated with 10 µM Rosiglitazone for 2 weeks and sampled 2 weeks post-laser ablation. There is much less αSMA staining in the ablation zone – thin, faint bands are visible, separated by zones devoid of αSMA staining, but not of PI-positive nuclei. This suggests that stromal cells are present, at what seems to be a fairly normal density, but that they are not myofibroblasts. The epithelium is also fully regenerated and well-organized. **F.** Untreated cat cornea 4 weeks post-laser ablation showing a much thinner, but still continuous band of αSMA staining below the ablation zone epithelium, which is now slightly thicker than at 2 weeks post-op, but still not quite normally organized. **G.** Cat cornea treated with Celluvisc vehicle solution, sampled at 4 weeks post-laser ablation. Note the distinct band of strong, continuous αSMA staining in the anterior stroma. The epithelium in this section was accidentally torn off and thus appears thinner than it actually was. **H.** Central ablation zone of a cat cornea treated with DMSO/Celluvisc vehicle solution for 2 weeks and sampled 4 weeks post-laser ablation. Again, there is strong, continuous αSMA staining in the anterior stroma, just below the epithelium, which is remarkably thickened relative to the unoperated condition (and normal corneas). **I.** Cat cornea treated with 10 µM Rosiglitazone in DMSO/Celluvisc for 2 weeks after PRK, and sampled at 4 weeks post-laser ablation. Just as in the 2 weeks condition for this treatment group, there was very little αSMA staining, organized into thin, faint clusters of αSMA positive cells separated by zones devoid of αSMA staining, but not of PI-positive nuclei. The epithelium is thick and well-organized.

Cat eyes in this study healed quickly, with epithelial regrowth over the denuded stromal surface complete between the 3^rd^ and 5^th^ days post-operatively. Normal, un-operated cat corneas exhibited a complete absence of αSMA staining within the stroma (**Fig. 1A**). In contrast, 2 and 4 weeks post-operatively, the ablation zone in all lasered eyes could be identified by a thickened, overlying epithelium (**Fig. 1**). However, there were clear differences in αSMA immunoreactivity between untreated, vehicle-treated (DMSO/Celluvisc and Celluvisc) and Rosiglitazone-treated eyes. Both untreated and vehicle-treated eyes exhibited a strong, continuous band of αSMA expression below the epithelium (**Figs. 1B, C, D**), which was thicker in untreated eyes at 2 weeks post-PRK (**Fig. 1B**), and essentially identical in all control groups at 4 weeks post-PRK (**Figs. 1F, G, H**). In eyes treated with 10 µM Rosiglitazone, only small, thin patches of αSMA were visible under the epithelium, alternating with αSMA-negative zones (**Fig. 1E, I**). Generally, αSMA expression was strongest at 2 weeks post-PRK, decreasing at 4 weeks (**Fig. 1**) and disappearing completely by 8 and 12 weeks after PRK, as previously reported for cat eyes undergoing this exact PRK treatment [27]. At 2 weeks post-PRK, Celluvisc and DMSO/Celluvisc treatments significantly reduced αSMA staining relative to the untreated corneas (contrast **Figs. 1B** with **1C, D**). This is consistent with the previously reported anti-inflammatory actions of both Celluvisc and DMSO [73], [74], [75]. However, by 4 weeks post-PRK, any qualitative differences between the thickness or intensity of the αSMA bands in untreated *versus* vehicle-treated controls had disappeared (contrast **Figs. 1F** with **1G, H**). At both time-points, there was markedly less αSMA-positive staining in Rosiglitazone-treated eyes than in untreated or vehicle-treated corneas. Thus, by 4 weeks post-PRK, neither DMSO, nor Celluvisc appeared to significantly influence αSMA expression – only Rosiglitazone did.

### Topical Rosiglitazone Decreases Corneal Haze after Laser Ablation

**Figure 2:**
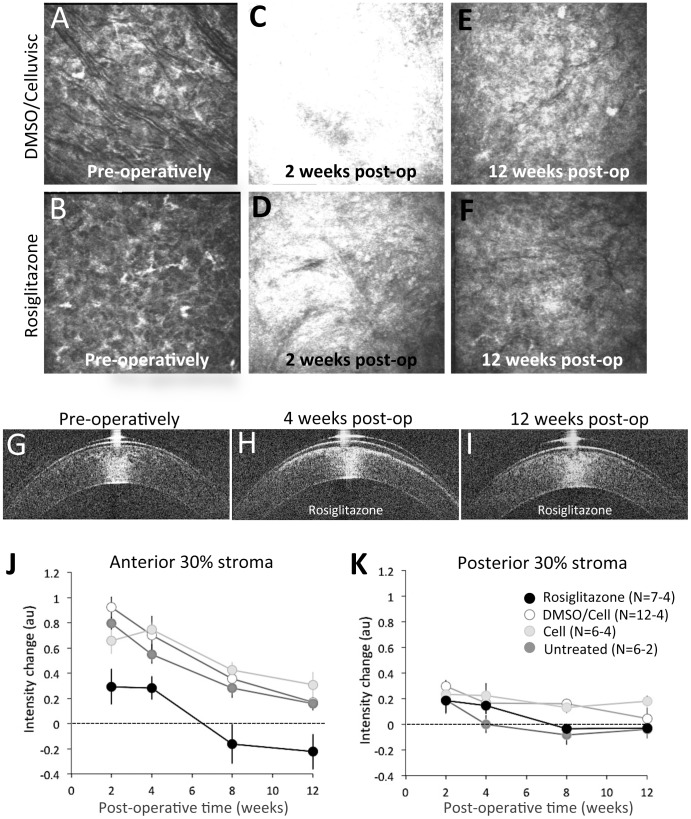
Optical imaging and the impact of Rosiglitazone treatment on wound healing in the cat cornea. Illustrative confocal images were taken 15 µm below the basal epithelial layer of the central cornea in one eye treated with DMSO/Celluvisc vehicle solution (**A, C, E**), and one eye treated with 10 µM Rosiglitazone (**B, D, F**) daily for 2 weeks after laser ablation. Pre-operative images (**A, B**) show a quiescent stroma that appears quite similar across the two eyes. Two weeks after laser ablation, just after the end of topical treatments, both corneas showed increased reflectivity, which was highest in the vehicle-treated eye (**C**) and least in the Rosiglitazone-treated eye (**D**). By 12 weeks post-operatively, the Rosiglitazone-treated eye’s reflectivity was back down to basal levels (**F**), while the vehicle-treated eye remained hazier than pre-operatively (**E**). OCT images of a Rosiglitazone-treated eye collected pre-operatively (**G**), 4 weeks (**H**) and 12 weeks (**I**) post-operatively illustrate a bright zone of reflectivity at 4 weeks post-op, which was not present pre-operatively and is lost by 12 weeks post-op. **J**. Plot of normalized intensity change in the anterior 30% of the stroma relative to pre-operative values (dotted line at zero), measured from OCT images such as those in G–I. **K**. Similar plot of normalized pixel intensity change against post-operative time for the posterior 30% of the stroma in either untreated, vehicle-treated or Rosiglitazone-treated cat corneas post-excimer laser ablation. Error bars = SEM. N = number of eyes, which differs at different time-points (see Table 1). DMSO/Cell = DMSO/Celluvisc treated eyes. Cell = Celluvisc treated eyes.

Pre-operatively, *in vivo* confocal imaging (**Fig. 2A, B**) and OCT imaging (**Fig. 2G**) showed the corneal stroma to have relatively low reflectivity. Two and 4 weeks post-operatively, both imaging modalities showed dramatically increased reflectivity in the ablation zone stroma (see **Figs. 2C, D** for confocal images; **Fig. 2H** for OCT images), which decreased back towards normal levels by 12 weeks post-operatively (**Fig. 2E, F** for confocal; **Fig. 2I** for OCT). However, there were significant differences between eyes treated with Rosiglitazone and those that were exposed to vehicle solutions:

Two and 4 weeks post-operatively, OCT backscatter reflectivity in the anterior 30% of the stroma of DMSO/Celluvisc-treated eyes had increased by 52±6.2% (mean±SEM) relative to pre-operative levels, decreasing thereafter, until 12 weeks post-operatively, when it was only 10±2.3% greater than pre-operative levels (**Fig. 2J**). A very similar pattern of results was obtained for cat eyes treated with Celluvisc and for untreated cat eyes. Rosiglitazone-treated eyes exhibited only a small (15±7.4%) increase in backscatter reflectivity in the anterior stroma relative to that seen in vehicle-treated and untreated eyes at the 2 and 4 weeks post-operative time-points (black symbols in **Fig. 2J**). A two-way ANOVA was conducted to examine the effect of post-operative treatment (Rosiglitazone or DMSO/Celluvisc vehicle) and time (2, 4, 8 and 12 weeks) on backscatter reflectivity in the anterior 30% of the stroma. There were significant main effects of treatment (F(1,41) = 26.12, p<0.0005) and time (F(3,41) = 11.349, p<0.0005) on reflectivity, but no significant interaction between the two (F(3, 41) = 0.609, p = 0.613). A similar, two-way ANOVA was conducted to assess whether there were significant differences between the 3 types of controls (DMSO/Celluvisc, Celluvisc and untreated eyes) over time (2, 4, 8 and 12 weeks) in terms of reflectivity of the anterior 30% of the stroma. We found no significant main effect of control type (F(2,50) = 0.015, p = 0.985) and no significant interaction between control type and time (F(6,50) = 1.069, p = 0.394). The only significant main effect was for time (F(3,50) = 13.993, p<0.0005), with reflectivity decreasing significantly between 2 and 12 weeks post-operatively in all 3 control groups.

As illustrated in **Figure 2K**, when combining all operated corneas at 2 weeks post-PRK, backscatter reflectivity of the posterior 30% of the stroma was greater than pre-operatively (p = 0.002, Student’s t-test). A two-way ANOVA was also conducted to examine the effect of post-operative treatment (Rosiglitazone versus DMSO/Celluvisc vehicle) and time (2, 4, 8 and 12 weeks) on backscatter reflectivity in the posterior 30% of the stroma. There were significant main effects of treatment (F(1,40) = 4.702, p = 0.036) and time (F(3,40) = 4.518, p<0.008) on reflectivity, but no significant interaction between the two (F(3, 40) = 0.568, p = 0.639). Post-hoc, Bonferroni-corrected t-tests between these two treatment groups at individual time-points revealed that the main significant difference in reflectivity between Rosiglitazone and DMSO/Celluvisc treated eyes occurred at 2 weeks post-PRK (p = 0.035). The reflectivity of the posterior stroma remained higher than baseline at all time-points for Celluvisc treated eyes, and until 12 weeks post-PRK, when it returned to basal levels in DMSO/Celluvisc-treated eyes (**Fig. 2I**). In contrast, untreated eyes exhibited a return to baseline as early as 4 weeks post-operatively (**Fig. 2I**).

### Rosiglitazone does not Impede Epithelial and Stromal Re-thickening after Injury

Pre-operatively, the central stroma was 460–600 µm thick, and the central epithelium 43–77 µm thick with no significant inter-group differences between eyes destined for the control *versus* treatment (Rosiglitazone) groups post-laser ablation (p = 0.9022, Student’s t-test).

**Figure 3:**
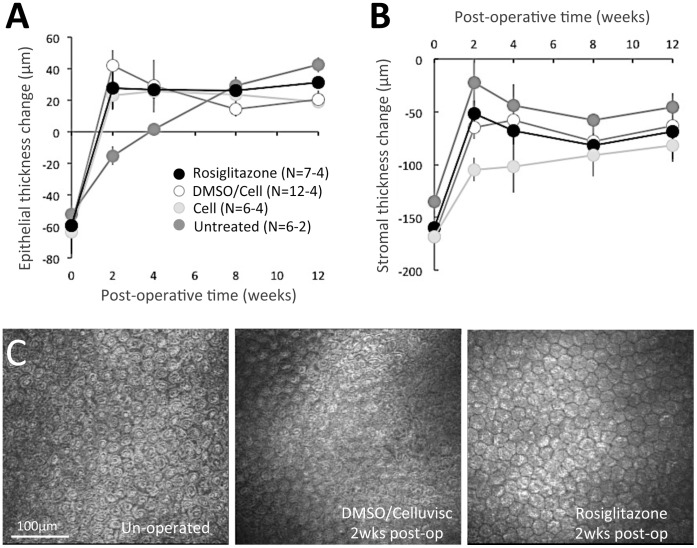
Effect of post-operative treatments on stromal and epithelial thickness. **A.** Plot of change in central epithelial thickness in the 4 experimental groups showing the average amount of epithelial thickness scraped off prior to laser ablation (time-point 0) and the hyperplasia in this layer that was already evident 2 weeks later in all but the untreated eyes, remaining relatively stable thereafter. By 8 weeks post-PRK, there is no longer a significant difference between experimental groups. **B.** Plot of change in central stromal thickness averaged across eyes in the 4 experimental groups, showing the amount of stromal thickness removed by laser ablation (at time-point 0) and the significant stromal thickening observed as soon as 2 weeks later in all groups (albeit to different extents). No significant differences were noted between groups after 4 weeks post-PRK. Error bars = SEM. N = number of eyes, which differs at different time-points (see Table 1). DMSO/Cell = DMSO/Celluvisc treated eyes. Cell = Celluvisc treated eyes. **C.** In vivo confocal imaging of the endothelial layer pre-operatively and 2 weeks post-laser ablation (at the end of topical treatment) in one DMSO/Celluvisc-treated cat eye and one eye that had received 10 µM Rosiglitazone post-operatively. Note tightly packed, mostly hexagonal cells, with no gaps between them, which are evident in both treatment groups.

The injury model used in the present study removed the entire epithelial thickness and an average 161±2.3 µm of stroma in the central cornea across all eyes, as verified intra-operatively with a Corneo-Gage Plus 2 ultrasonic pachymeter (Sonogage Inc.). Two weeks later (at the end of pharmacological treatment), OCT measurements showed that the epithelium had regenerated and was in fact thicker than pre-operatively by an average of 30 µm in vehicle and Rosiglitazone-treated eyes, but not in untreated eyes (**Fig. 3A**). A two-way ANOVA was conducted to examine the effect of post-operative treatment (Rosiglitazone, DMSO/Celluvisc vehicle, Celluvisc vehicle and no treatment) and time (0, 2, 4, 8 and 12 weeks) on epithelial thickness. There were significant main effects of treatment (F(3,117) = 9.130, p<0.0005) and time (F(4,117) = 5.736, p<0.0005), as well as a significant interaction between the two (F(12, 117) = 2.815, p<0.0005). Bonferroni-corrected post-hoc t-tests showed the untreated group to behave significantly differently from all other groups in terms of epithelial thickness. When comparing the 2 week epithelial thickness of each individual group with their pre-operative values, both Rosiglitazone and vehicle-treated corneas had significantly thicker epithelia than pre-operatively (p = 0.04 for Rosiglitazone, p = 0.01 for Celluvisc, p = 0.003 for DMSO/Celluvisc-treated eyes - **Fig. 3A**). However, untreated control eyes exhibited delayed epithelial regeneration, only catching up to the treated eyes (whether Rosiglitazone or vehicle) by the 8^th^ post-operative week (**Fig. 3A**). At 2 weeks post-PRK, untreated corneas exhibited significantly thinner epithelia than pre-operatively (p = 0.009, Student’s t-test). It took until the 4^th^ post-operative week for untreated epithelia to return to pre-operative central thickness values (p = 0.2887, Student’s t-test).

Central stromal thickness also increased after the ablation, though it remained thinner than pre-operatively in all groups 12 weeks post-operatively (**Fig. 3B**). A two-way ANOVA was used to examine the effect of post-operative treatment (Rosiglitazone, DMSO/Celluvisc vehicle, Celluvisc vehicle and no treatment) and time (0, 2, 4, 8 and 12 weeks) on stromal thickness. There was no significant main effect of treatment (F(3,117) = 2.441, p = 0.068) - only of time (F(4,117) = 4.155, p = 0.003), and a marginally significant interaction between the two (F(12, 117) = 1.870, p = 0.045). There appeared to be delayed stromal thickening in the Celluvisc-treated eyes relative to the others at 2 weeks post-PRK. However, this difference disappeared at later time-points. By the 12th post-operative week, there were no longer any significant differences in stromal thickness between treatment groups. However, *p*<0.05 relative to pre-operative levels in each group (p = 0.027 for Rosiglitazone, p = 0.0002 for DMSO/Celluvisc, p = 0.007 for Celluvisc and p = 0.027 for untreated eyes, Student’s t-tests). Central epithelial and stromal thicknesses remained relatively stable from 4 to 12 weeks post-laser ablation, with no significant inter-group differences.

Whether the observed stromal thickness change reflected regeneration or swelling is a matter of contention. At the latest post-operative time-points, however, the hypothesis that it may have represented regeneration was supported by 3 observations: (1) OCT data (**Fig. 2**) showing that corneal haze was minimal (in control eyes) or absent (in Rosiglitazone-treated eyes) at 12 weeks post-op; (2) the epithelial layer was thick and well organized; and (3) the central endothelium was normal in appearance and density (**Fig. 3C**). The mean±SEM pre-operative endothelial cell density was 2120±86 cells/mm^2^ and 2140±150 cells/mm^2^ for eyes destined for vehicle and Rosiglitazone treatments, respectively. Eight to 12 weeks post-laser ablation, central endothelial density was 1788±213 cells/mm^2^ in DMSO/Celluvisc-treated eyes and 1912±97 cells/mm^2^ in Rosiglitazone-treated eyes. Neither number was significantly different from pre-operative levels (p = 0.1253 for DMSO/Celluvisc and p = 0.3711 for Rosiglitazone, Student’s t-tests) or from each other (p = 0.5814, Student’s t-test).

### Rosiglitazone Minimizes Defocus Change and Residual Higher Order Aberration Induction

**Figure 4:**
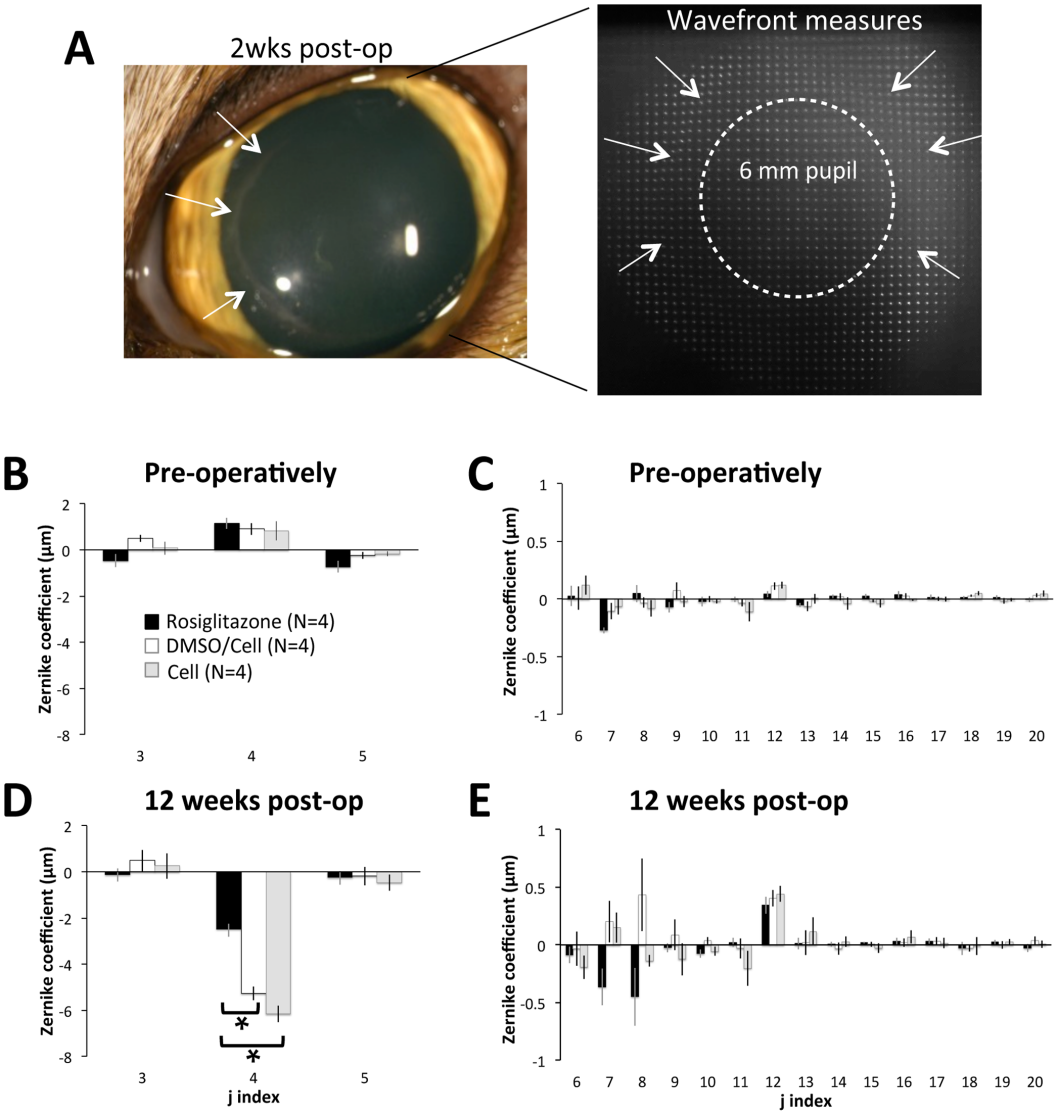
Impact of laser ablation and Rosiglitazone treatment on the magnitude of ocular wavefront aberrations. **A.** Picture of a DMSO/Celluvisc-treated cat eye 2 weeks after surgery, illustrating ocular appearance and the quality of the spot array patterns imaged with the wavefront sensor at this time-point. Note the haziness of spots at this time-point, as well as the distortion clearly visible at the edge of the laser ablation on the cornea and the spot array pattern**.** The dashed white circle denotes the 6 mm diameter optical zone over which wavefront aberrations in the graphs below (B–E) were quantified. **B**. Plots of mean pre-operative 2^nd^ order aberrations (j = 3–5) in the 3 treatment groups (Rosiglitazone-treated, DMSO/Celluvisc and Celluvisc), indicating near emmetropia in all cases. **C.** Pre-operative magnitude of higher order aberrations (HOAs) up to the 5^th^ order (J = 6–20), showing no significant inter-group differences and relatively low HOAs over a 6 mm pupil. Note however, that primary coma (j = 7) and spherical aberration (j = 12) were the dominant HOAs in all groups. **D.** Mean lower order aberrations over a 6 mm pupil 12 weeks post-PRK showing virtually no change in astigmatism terms (j = 3 and 5) but a substantial myopic shift in the defocus term (j = 4), which was significantly greater for vehicle-treated eyes than in those treated with Rosiglitazone post-operatively. **E.** Plot of mean HOAs 12 weeks post-PRK showing a progression towards more negative primary coma terms (j = 7 and 8) for Rosiglitazone-treated eyes, while primary coma terms in vehicle-treated eyes were more positive, on average. Spherical aberration (j = 12) became more positive in all groups, by about the same amount. Error bars = SEM, N = number of eyes, * p<0.05, Student’s t-test for differences relative to Rosiglitazone-treated eyes.

Pre-operatively, cat eyes were slightly myopic, hovering around 1 µm of positive defocus over a 6 mm pupil (corresponding to about −0.9 D), with low astigmatism (**Fig. 4B**). Higher order aberrations (HOAs) were very small, with vertical coma (j = 7) being the most significant (**Fig. 4C**). There were no significant differences in lower- or higher-order aberrations between cat eyes destined for vehicle *versus* Rosiglitazone treatment.

Post-operatively, the situation changed radically. By 12 weeks after surgery, after wound healing was complete and the corneas appeared to have stabilized biologically, significant differences were seen between vehicle-treated eyes (whether with DMSO/Celluvisc or Celluvisc – there were no significant differences between these two groups) and those that received Rosiglitazone post-laser ablation. While all eyes became more hyperopic, eyes that received vehicle solutions ended up with −5.2±0.4 µm and −6.2±0.4 µm of defocus over a 6 mm-diameter optical zone for DMSO/Celluvisc and Celluvisc-treated eyes respectively (white and grey bars in **Fig. 4D**), which represents about a 5–6D change relative to pre-operative values. In contrast, eyes treated with Rosiglitazone had only −2.5±0.3 µm of defocus over a 6 mm diameter zone (black bars in **Fig. 4D**), a change of only 2.8D relative to pre-operative values. As expected given the Planoscan ablations used, astigmatism terms remained unchanged (close to zero) in both groups (**Fig. 4B**). Thus, at the end of the 12 week post-surgical observation period used in this experiment, Rosiglitazone-treated eyes were closer to emmetropia by a factor of 2 relative to vehicle-treated eyes. A two-way repeated measures ANOVA with treatment group as the between-subject factor and time as the within-subject factor revealed a significant main effect of time (F(2,10) = 15.621, p = 0.001) and a significant interaction between treatment group and time (F(2,10) = 5.804, p = 0.021). By 12 weeks post-laser ablation, LORMS change was significantly greater in DMSO/Celluvisc-treated than in Rosiglitazone-treated eyes (p = 0.0008, Student’s t-test). In contrast, astigmatism RMS did not change from pre-operative values for any of the treatment groups, at any of the time-points examined.

**Figure 5:**
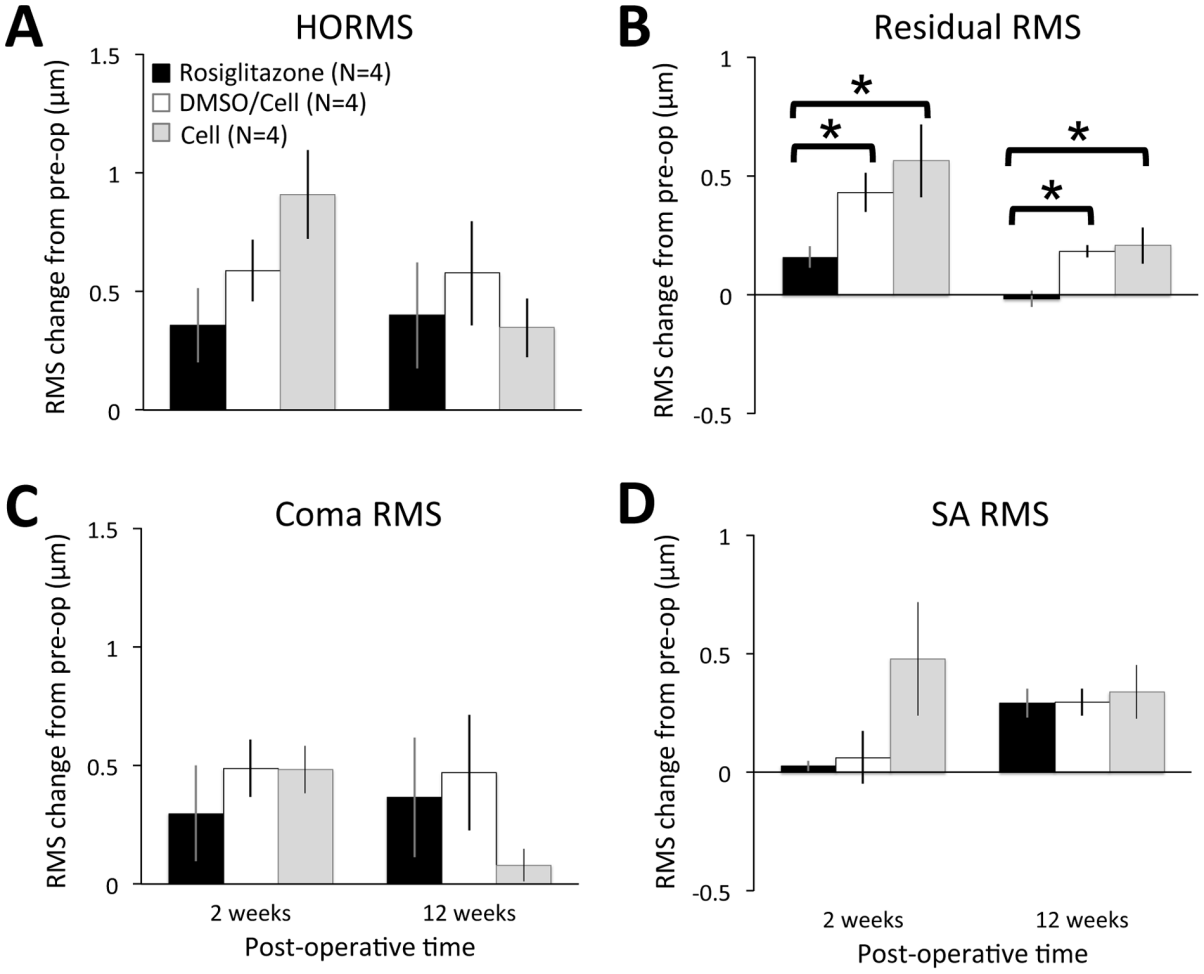
Change in higher order wavefront aberration root mean square (RMS) 2 and 12 weeks after laser ablation relative to pre-operative levels. **A.** Plot of change in higher order RMS (HORMS) at 2 and 12 weeks post-laser ablation in Rosiglitazone-, DMSO/Celluvisc and Celluvisc -treated cat eyes. HORMS increased significantly relative to pre-operative levels in all groups of eyes, and this elevation was maintained all the way out to 12 weeks post-PRK, but there were no significant inter-group differences in the magnitude of the change, whose significance was likely driven predominantly by spherical aberration (SA). **B.** Plot of change in residual HORMS illustrating significantly smaller increases in Rosiglitazone-treated eyes relative to both vehicle-treated eyes. These differences are maintained out to 12 weeks post-operatively. **C.** In contrast, the magnitude of increase in coma RMS is not significantly different between treatment groups, at either 2 or 12 weeks post-PRK. **D.** Finally, spherical aberration (SA) RMS does not appear significantly increased 2 weeks post-laser ablation in Rosiglitazone or DMSO/Celluvisc-treated eyes, but all 3 groups show a similar, positive change in SA RMS relative to pre-operative values by 12 weeks post-PRK. Error bars = SEM, N = number of eyes, * p<0.05, Student’s t-test.

A cursory comparison between plots in **Figs. 4C and E** shows that some higher order aberrations were clearly altered in both vehicle- and Rosiglitazone-treated eyes. The results of RMS analysis, used to tease out some of the more subtle differences in HOA changes, are as follows: prior to laser ablation, HORMS in eyes destined for Rosiglitazone treatment was around 0.42±0.03 µm. Residual (non-coma, non-spherical) HOAs accounted for most (49±5%) of the HO variance, followed by coma-like aberrations (39±7%), then spherical-like aberrations (10±4%). There were no statistically significant differences in any of the terms examined between the 3 groups. Post-operatively, some HOAs (**Fig. 4E**) and HORMS (**Fig. 5A**) increased in both Rosiglitazone and vehicle-treated eyes. However, the nature of, the temporal pattern of induction, and the persistence of these aberrations differed significantly between Rosiglitazone and vehicle-treated eyes. In particular, there were two interesting observations: first, there was a general delay in HOA induction in Rosiglitazone-treated eyes *versus* those receiving vehicle after the laser ablation, with increases in HORMS, coma RMS and residual RMS first significant at 2 weeks for DMSO/Celluvisc and Celluvisc-treated eyes, and at 4 weeks for Rosiglitazone-treated eyes. The second interesting observation was that by the 12^th^ post-operative week, in Rosiglitazone-treated eyes all HOAs had decreased back to levels not significantly different from pre-operative values, except for spherical aberration RMS, which remained significantly elevated relative to pre-operative values (**Fig. 5D**). In vehicle-treated eyes, all HOAs remained elevated, except for coma RMS, which decreased back to normal (**Fig. 5**). As an illustration, by 12 weeks post-laser ablation, residual HOAs only accounted for 15±6% of the HO variance in Rosiglitazone-treated eyes, a significant decrease even relative to pre-operative levels (p = 0.019, Student’s t-test – **Fig. 5B**). In DMSO/Celluvisc and Celluvisc vehicle-treated eyes, these irregular, fine-grained HOAs still accounted for 35±13% and 46±3% of the HO variance at 12 weeks, respectively (**Fig. 5B**). In contrast, no statistically significant differences in the magnitude of induced SA were observed between the treatment groups at 12 weeks (**Fig. 5D**). This was verified with a two-way ANOVA that probed the effects of post-operative treatment (Rosiglitazone, DMSO/Celluvisc vehicle or Celluvisc vehicle) and time (2 and 12 weeks) on SA RMS change. There was no significant main effect of treatment (F(2,18) = 3.289, p = 0.061) or time (F(1,18) = 1.129, p = 0.302), and there was no significant interaction between the two (F2, 18) = 2.053, p = 0.157).

In summary, vehicle-treated eyes tended to show a rapid induction of the main HOAs following PRK, and all but coma-like aberrations were maintained long-term (i.e., to 12 weeks post-PRK). The only apparent contribution of DMSO to the effect of these vehicle solutions was to delay the increase in spherical-like HOAs until 4 weeks post-PRK (**Fig. 5D**). Rosiglitazone-treated eyes experienced a delayed induction of *all main classes of HOAs*, and out of these, only spherical-like HOAs persisted out to 12 weeks post-PRK.

### Rosiglitazone Blocks TGFβ-induced αSMA Expression in Cultured Feline Corneal Fibroblasts

**Figure 6:**
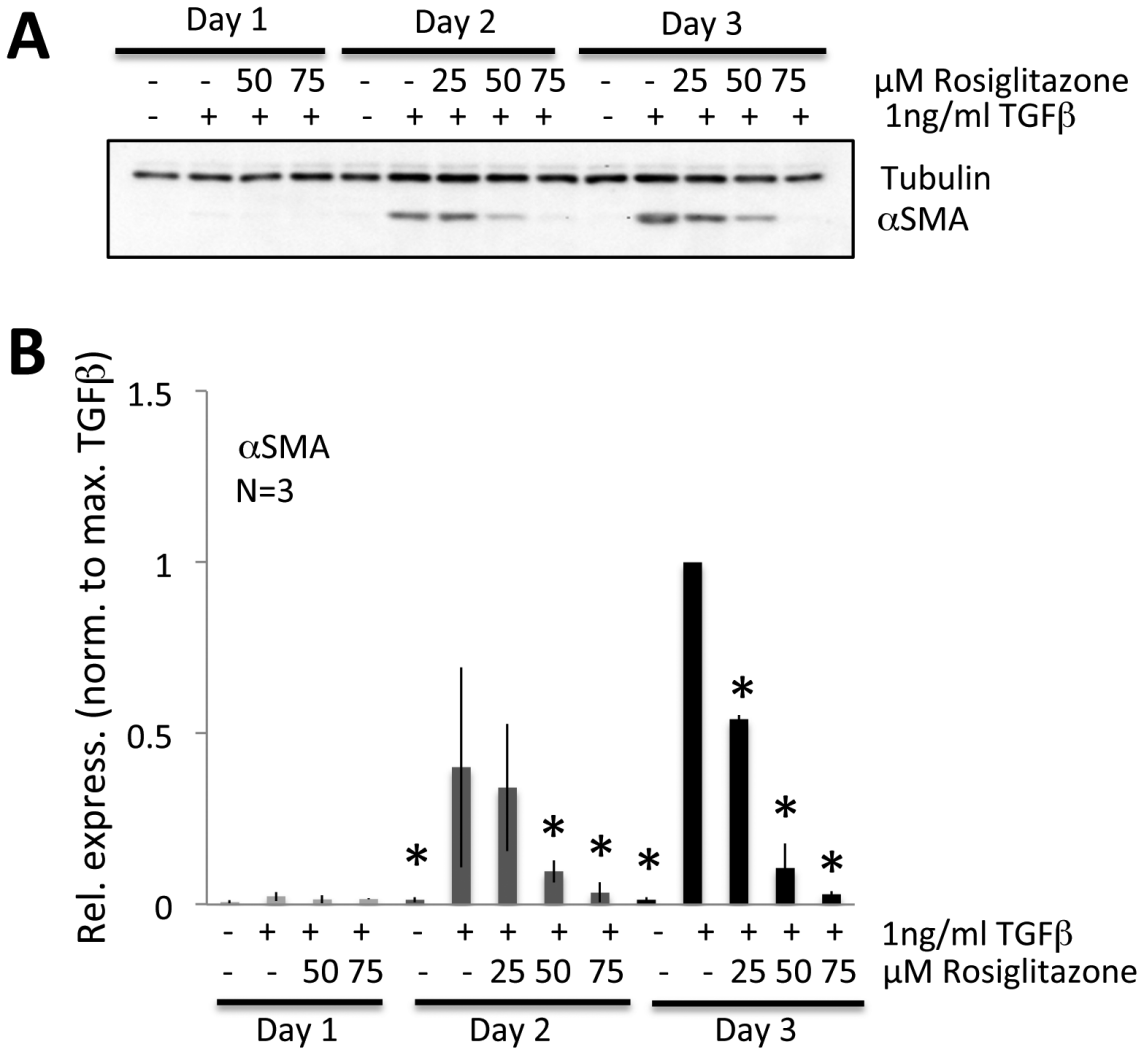
Anti-fibrotic effects of Rosiglitazone on cultured feline corneal fibroblast. **A**. Representative western blots showing protein levels for αSMA. Tubulin levels were assayed as a loading control. For this experiment, cells were pretreated with 25 µM, 50 µM, and 75 µM Rosiglitazone for 30 min, before adding 1 ng/ml of TGFβ in DMEM/F12 containing 1% HS. The cells were cultured in this treated medium for 1, 2 or 3 days and then harvested for western blotting. While some effect could be observed at lower doses, Rosiglitazone clearly inhibited αSMA expression at 75 µM, while tubulin levels remained stable. **B**. Plots of relative expression of αSMA normalized to densitometric values obtained in cells stimulated with 1 ng/ml TGFβ for each culture day sampled. Data shown are means±SD, averaged over 3 experiments, and they confirm a statistically significant inhibitory effect of Rosiglitazone on αSMA. * P<0.05, Student’s t-test *relative to TGFβ-only condition*.

Our *in vivo* results suggested that topical application of Rosiglitazone to the injured cat cornea inhibited myofibroblast differentiation. To verify whether Rosiglitazone exerted this effect directly on stromal fibroblasts, primary feline corneal fibroblasts were cultured and pre-treated with different doses of Rosiglitazone before being stimulated by the addition of TGFβ. Western blotting performed on a sample of the cells after 1, 2 and 3 days in culture revealed that in the absence of TGFβ stimulation, basal levels of αSMA expression were close to zero even after 3 days in culture (**Figs. 6A, B**). It then took 3 days for αSMA expression to peak after TGFβ stimulation, but the strongest dose of Rosiglitazone that could be used without inducing toxicity (75 µM) decreased αSMA expression after 2 and 3 days in culture (**Fig. 6A,B**). After 3 days in culture, treatment with 75 µM Rosiglitazone reduced αSMA expression relative to levels induced by 1 ng/ml TGFβ by between 79 and 98% (mean±SD = 90±8%). A two-way ANOVA that probed the effects of treatment (control, 1 ng/ml TGFβ or 75 µM Rosiglitazone) and time (1, 2 and 3 days in culture) on αSMA expression showed a significant main effect of treatment (F(2,18) = 34.226, p<0.0005), time (F(2,18) = 19.098, p<0.0005), and there was also a significant interaction between the two (F4, 18) = 16.600, p<0.0005). Post-hoc Bonferroni corrected t-tests showed that significant effects between treatments across time resided only in the TGFβ-treated cells. There were no significant differences in αSMA expression between control and 75 µM Rosiglitazone treatment groups across time.

## Discussion

According to the World Health Organization, corneal scarring is the second major cause of blindness worldwide behind cataracts [76]. Over the last 25 years, significant progress has been made in understanding the cellular and molecular bases of corneal fibrosis, the precursor to scarring. However, we cannot yet effectively control fibrosis in the cornea or elsewhere in the body. Here, we show that topical administration of Rosiglitazone to the eye can effectively block myofibroblast differentiation, a hallmark of corneal fibrosis, while allowing the epithelium and stroma to return to a normal thickness, restoring corneal shape, structure and optical quality to near-normal levels. Cell culture experiments confirmed the anti-fibrotic effects of Rosiglitazone on isolated corneal fibroblasts and revealed a significant impact of Rosiglitazone on αSMA expression.

### Rosiglitazone Blocks TGFβ-induced Expression of αSMA in the Cornea *in vivo* and *in vitro*

Excimer laser ablation of the kind performed here (i.e. PRK for high myopia) is known to generate a strongly-stained band of αSMA expression in the sub-epithelial stroma of a range of species, including humans, rabbits and cats [27], [32], [60], [77], [78], [79]. Generally, this band becomes visible 1 week post-PRK, increasing in intensity by 2 weeks post-PRK, and decreasing thereafter until it disappears between weeks 8 and 12 [27], [32]. This phenomenon was confirmed in vehicle-treated and untreated eyes in the present study. Interestingly, in spite of DMSO being a known anti-inflammatory agent [73] and the carboxymethylcellulose in Celluvisc actively promoting epithelial wound healing in the cornea [74], [75], these two vehicle solutions only dampened, rather than eliminated αSMA expression relative to untreated eyes. Only the presence of Rosiglitazone effectively blocked stromal αSMA expression, with only a few, interspersed patches of myofibroblasts observed 2 and 4 weeks post-PRK and no detectable staining at 8 and 12 weeks. Just as with anti-TGFβ treatment in two prior studies [27], [80], αSMA expression did not suddenly increase after daily anti-fibrotic treatment was discontinued, suggesting that early inhibition of myofibroblast differentiation may be sufficient for long-term fibrosis control after excimer laser ablation of the stromal surface. An important question that remains to be answered is whether starting application of Rosiglitazone at later time-points post-injury is able to prevent or reverse the scarring process. Finally, consistent with the effectiveness of Rosiglitazone at blocking αSMA expression *in vivo*, this PPARγ ligand also reduced αSMA expression in cultured, feline, corneal fibroblasts stimulated with TGFβ.

### Topical Rosiglitazone Decreases post-operative Haze in the Anterior Corneal Stroma

In Rosiglitazone-treated cat eyes, low αSMA expression was accompanied by a significantly decrease in laser-induced haze in the anterior stroma, as measured by OCT backscatter reflectivity and confirmed qualitatively with confocal imaging. By 8 and 12 weeks post-PRK, anterior stromal reflectivity in Rosiglitazone-treated corneas was indistinguishable from pre-operative levels, and significantly lower than in vehicle-treated and untreated corneas. It is possible that low levels of light scatter were caused by a reduction in cellular repopulation of the injured tissue (due to drug toxicity, decreased proliferation, etc.). Although qualitative inspection of histological sections stained with propidium iodide (a nuclear stain) did not indicate that this was occurring in our samples, cell counts and further analysis would be required to completely rule this out. However, assuming that decreased cell density does not account for the lack of haze observed presently, the remarkable implication here is that by 2 months post-laser ablation, from an optical clarity stand-point, Rosiglitazone-treated corneas looked macroscopically like they had never been operated upon. Importantly, post-operative treatment with just DMSO and/or Celluvisc did not significantly improve corneal haze relative to untreated corneas post-PRK.

### Rosiglitazone Treatment does not Block Corneal Thickening after Laser Ablation

Perhaps one of the most striking observations in the present study was the speed with which the epithelial and stromal thicknesses increased following the laser ablations in all treatment groups. Significant thickening of the epithelium above pre-operative levels occurred in Rosiglitazone and vehicle-treated eyes by 2 weeks, and in untreated eyes by 8 weeks post-operatively. The delayed epithelial thickening in untreated eyes is consistent with the fact that these were the only eyes not receiving Celluvisc-containing eye drops post-PRK. As mentioned earlier, Celluvisc is known to promote epithelial healing in the cornea [74], [75]. Regardless, by 12 weeks post-operatively, all eyes in the present study exhibited central epithelium that was about 30 µm thicker on average than pre-operatively. This was a very different outcome than obtained previously with anti-TGFβ treatment post-PRK in the cat [27], and also in rabbits [81], [82] where epithelial thickness eventually returned to normal rather than exhibiting hyperplasia. A possible explanation for this difference is that the prior studies used much shorter treatment periods than in the present experiments. The other possible explanation is that neutralizing antibodies to TGFβ are known to inhibit epithelial regrowth in corneal organ culture and in epithelial wound healing studies in mice [83], [84]. It is thus conceivable that even when anti-TGFβ antibodies were mixed with Celluvisc in prior studies [27], [80], the epithelial growth-promoting effects of Celluvisc were likely insufficient to completely counteract the inhibition enacted by anti-TGFβ antibodies on epithelial cells. In the present experiments, Rosiglitazone was administered with both DMSO and Celluvisc. In addition to the epithelial-growth promoting effects of Celluvisc, DMSO is an effective anti-ulceration agent in skin [85], [86] and could play a similar role in the cornea. Ultimately, when Rosiglitazone was dissolved in DMSO/Celluvisc, the compound solution did not inhibit re-epithelialization. Whether this was because Rosiglitazone did not inhibit the SMAD pathway in TGFβ signaling, and/or because Rosiglitazone allowed phosphorylation of p38-MAPK and its translocation to the nucleus, which appears to be necessary for epithelial resurfacing [83], remains to be determined. Either way, fast epithelial re-growth is highly desirable for corneal wound healing. An open epithelium represents an irritation to the corneal surface that prolongs secretion of TGFβ, which in turn, exacerbates the intensity of the fibrotic response in stromal keratocytes [87], [88]. Clearly, this was avoided in corneas treated with Rosiglitazone/DMSO/Celluvisc post-PRK.

The central stroma of vehicle-treated, Rosiglitazone-treated and untreated cat eyes also thickened significantly by 2 weeks post-laser ablation. In contrast, the stroma of eyes receiving anti-TGFβ treatment for 2 weeks post-PRK remained ∼160 µm thinner than normal, even as long as 3 months post-operatively [27]. Significant stromal regeneration was previously observed in rabbit models of PRK [80], [81], [82], although in that species, it appeared to take a long time (closer to 6 months). Of note, however, is the fact that short-term anti-TGFβ treatment in the rabbit did not inhibit stromal regeneration [80]. Together with the present Rosiglitazone effects, this may suggest that aspects of stromal regeneration could be regulated via TGFβ-independent pathways.

In addition, the possibility that the post-operative increase in corneal thickness observed in our *in vivo* experiments was due to swelling remains, especially at early time-points post-PRK. Increased water infiltration into the stroma, resulting in swelling, could occur in two main ways – a leaky endothelium and a leaky epithelium. However, confocal imaging revealed an endothelium that looked normal and unstressed at all time-points, with no polymegathism or cell vacuolization. Though limited to sampling of the central cornea, analysis of *in vivo* confocal images showed no significant changes in endothelial cell density across post-operative time-points. Thus, swelling as a result of endothelial failure was unlikely. At later time-points, the epithelium was thick (in fact, thicker than normal – see Fig. 1), well stratified and unlikely to have been leaky, although this was not explicitly tested in the present study. Nevertheless, corneal swelling is normally accompanied by haze, yet by 8 and 12 weeks post-laser ablation, the OCT-based stromal reflectivity measures in Rosiglitazone-treated eyes indicated no haze in either the anterior or posterior thirds of the cornea. Thus, while we cannot rule out that the vehicle-treated and untreated corneas remained somewhat edematous throughout the experimental period, at least the Rosiglitazone-treated eyes did not appear to suffer from this problem.

### Rosiglitazone Restores Near-emmetropia Following Laser Ablation of the Cornea

Given that the cornea’s total thickness returned to within 20 µm of normal within 2 weeks after complete epithelial removal and laser ablation of the central stroma to a depth of ∼160 µm, we predicted that this would erase the wavefront aberration changes induced by the surgery. However, this only happened in Rosiglitazone-treated eyes. Vehicle-treated eyes exhibited about 5–6D of hyperopic shift and a significant, lasting increase in HOAs, including spherical aberration (SA) and residual HOAs – a result consistent with our previous data using -10D PRK over a 6 mm optical zone in cats [27]. Rosiglitazone-treated eyes exhibited less than half this defocus shift, going from 1D of myopia to 1D of hyperopia. Thus, in Rosiglitazone-treated eyes, the ablation shape was largely erased. Since myofibroblast differentiation was blocked only in Rosiglitazone-treated eyes (and not in any of the vehicle-treated eyes), we may conclude that blocking myofibroblast differentiation and the tissue contraction these cells would enable, together with a return to near-normal total corneal thickness worked together to bring corneal optics in Rosiglitazone-treated eyes back towards emmetropia. In addition, Rosiglitazone-treated eyes exhibited delayed induction of HOAs (by 2 weeks) relative to vehicle-treated eyes, and overall, they exhibited lower residual (non-coma, non-spherical) HOAs 12 weeks post-operatively. However, once induction of spherical-like HOAs was observed at 4 weeks post-PRK, it persisted until the end of the observation period, at 12 weeks post-PRK. Generally, our results with Rosiglitazone treatment appear consistent with our prior observations following anti-TGFβ treatment post-PRK in the same cat model, in which we showed decreased myofibroblast differentiation to be correlated with reduced residual HOA induction [27]. Another similarity between Rosiglitazone and anti-TGFβ treatment was that both ultimately resulted in a persistent increase in spherical aberration. However, with respect to HOAs, Rosiglitazone did exhibit a distinct advantage over anti-TGFβ treatment: in the latter, all reductions in HOAs observed were short-lived, being significant only at the 2 weeks time-point post-PRK. Here, we show that Rosiglitazone’s ability to reduce residual HOAs is maintained well beyond the 2 weeks post-operative period during which the drug was administered. Overall, whether the emmetropization effect of Rosiglitazone is truly permanent remains to be determined. Clearly, this drug is not optimal for use following laser refractive surgery, where refractive changes are intended and desirable. Instead, Rosiglitazone would most likely be highly beneficial in situations where a [pathological] corneal wound caused unwanted changes in refractive power and/or higher order aberrations, and where the patient’s visual recovery would actually depend on near-total restoration of corneal thickness as well as refractive power.

### Putative Mechanisms of Rosiglitazone’s Anti-fibrotic Actions in the Cornea

Like Pioglitazone, Troglitazone and Ciglitazone, Rosiglitazone belongs to the Thiazolidinedione (TZD) class of drugs, and has traditionally been used as an oral hypoglycemic agent for the treatment of Type II diabetes [89], [90]. While TZDs are effective insulin-sensitizers [91], they can also act as anti-inflammatory agents in such conditions as rheumatoid arthritis, inflammatory bowel disease, ischemia-reperfusion injury and experimental encephalomyelitis [38], [39], [92], [93], [94]. Finally, TZDs have been shown to act as anti-fibrotics in both lung [44] and corneal tissue [53] *in vitro*. However, while their ability to control lipid metabolism is PPARγ-dependent [46], [47], [48], accumulating evidence, suggests largely PPARγ-independent, off-target effects for controlling fibrosis. These could include impact on SMAD, Integrin/FAK, as well as p38/MAPK pathways, all of which can contribute to fibrosis under different circumstances (see recent review [95]). Ongoing cell culture experiments in our laboratory are examining the effects of PPARγ ligands on SMAD2/3 phosphorylation and nuclear translocation, as well as on phosphorylation and activity of Rho, Erk, JNK, p38 and Akt – all of which are known to be activated by TGFβ receptor binding in fibroblasts.

*In vivo*, stromal keratocytes are unlikely to be the only cells impacted by Rosiglitazone – both epithelial and inflammatory cells play important roles in corneal wound healing [96]. Thus, it is possible that some of the beneficial effect of Rosiglitazone in corneal wound healing involved direct action on epithelial and/or inflammatory cells [97], [98], and these processes may include both PPARγ-dependent and -independent signaling. Indeed, PPARγ-dependent signaling is already known to play a role in inflammation control (reviewed in [99]). Further studies are needed to examine the relative contribution of immune cell inhibition by this TZD to the anti-fibrotic effects observed in the present animal model. Similarly, the mechanisms of action of Rosiglitazone in epithelial cells need to be clarified. Diversity of TZD action in different cell types, and even of different TZDs in the same cell type, underscores a necessity to systematically detail their behavior and mechanisms of action in both damaged and un-operated, healthy corneas. Only with this knowledge, will we be able to properly control topical administration of Rosiglitazone and other TZDs and to optimize treatment outcomes in preparation for clinical implementation of this work.

In conclusion, Rosiglitazone inhibited the appearance of αSMA-positive cells in the wound area of excimer-laser-ablated cat corneas, but allowed rapid stromal and epithelial re-thickening to occur. Three months later, the result was a cornea whose total thickness had returned to near-normal, whose refraction had returned to near-emmetropia, and whose clarity had returned to normal. Clear differences emerged between Rosiglitazone and anti-TGFβ treatment following laser ablation in our cat model, confirming the hypothesis that Rosiglitazone likely exerted its anti-fibrotic actions in the cornea via pathways downstream of TGFβ signaling. Thus, it appears that manipulating pathways downstream of TGFβ signaling could be more beneficial in healing a corneal wound than neutralizing TGFβ.
